# Learning and other developmental disorders in India

**DOI:** 10.4103/0019-5545.69236

**Published:** 2010-01

**Authors:** Philip John

**Affiliations:** Peejays Child Guidance Clinic, Cochin, S. India and CGC, Sharjah, UAE

**Keywords:** Child Psychiatry, developmental disorders, Indian Journal of Psychiatry, learning disorders

## Abstract

Articles that include Learning and Developmental Disorders have been gathered from the Indian Journal of Psychiatry (IJP) archives, and are broadly discussed. Learning disorders (LD) are not pure syndromes. They are developmental disorders and are multi-dimensional in nature. Research areas in Child Psychiatry in India remain largely unexplored, especially developmental disorders. The potential for research is mind boggling. Original research must keep pace with work in the west, and must be of a high order. Results must be published in our national journal and not abroad, in order to bestow prestige to our journal, so the world can sit up and take notice.

## INTRODUCTION

“*Humans were not born to read, or to write*”.

(Rosemary Tannock)

Annotations are explanatory notes; the editor’s brief to me was to review articles pertaining to Learning Disorders that were published in the IJP. These have been abysmally few, yet many studies allude to these disorders. The passionate editorial by T.S.S. Rao and V.S.T. Krishna on “Stars on the Ground”[1] and the Clinical Practice Guidelines for Specific Learning Disorders by Nilesh Shah and Tushar Bhat”[2] are exceptions.

For obvious reasons, outstanding or original studies by psychiatrists done in India used to get published abroad or in various national journals; or, if they failed to replicate western thought, they did not get published at all.

We seem to live in reverential wonder of the western thinking (which may be fine), but often refuse to contemplate out of that box. More than a decade ago, some of us thought that Asperger’s syndrome may be a high-functioning autistic disorder, and later, that obsessive disorder in childhood, unlike adult obsessive compulsive disorder (OCD), may be developmental in origin. These thoughts could not then cross the peer-review barrier, as our fraternity was bound by western concepts; yet today we so willingly accept them! The absence of publication of similar original thinking and work in our Indian journal not only takes away the prestige of the journal, but also puts us a step behind the west every time.

For the amount of replications of western studies that we do, whether they are epidemiological methods or ‘standardizing’ instruments, such articles must not make us appear like the footslogger trying to catch up with the racing car.

### Do deliver old wine in new bottles, after appropriate research

We, in India, have been apologetic of examining our traditional concepts to present them as concrete paradigms for prevention or management of disorders. If cognitive behavior therapy can be a concrete management strategy, so can the doctrines of the Bhagawad Gita, provided we can formulate them as a concrete management strategy through appropriate research and publication. It is one of the most successful paradigms for restructuring cognition even during brief contact with patients, whether children on the eve of their exams, or adults after failing to get a coveted promotion. In Anxiety, or in Depression. The simplicity of its application, even for the patient whose metacognition is poor, is its true strength.

Or for that matter, look at our traditional Hierarchical Parenting strategies. Parenting by keeping a personal space from them teaches our children discipline without having to punish, tolerance without having to indulge and respect without having to yell or scream. Such a ‘hierarchical parenting package’ can build capacity in our children for tolerance to frustration, tolerance to criticism, and a tolerance to change; it can teach them the strength of internal discipline, which has been the traditional Indian expectation from parenting. However, such prudent parenting is today rebuked and ridiculed by many of us because it is not ‘stylish’, not acceptable to the metropolitanism of our media, and inimical to the western family percept! Instead of raving and rhapsodizing in the media about the non-hierarchical strategy of “father being a friend”, has any of our researchers undertaken to prove or disprove the value of hierarchical parenting without punishment, and offer it as a paradigm for inculcating discipline in children? (This is an opportune time to study the effectiveness of our hierarchical parenting and organization, and present it to the world, when the west is smarting under the downturn of their financial systems, due to lack of disciplinary governance).

All these concepts can become original studies from India, based on generations of their replicability, which is what science is about. I am certain these strategies can catch up as ‘packages’ like yoga and meditation, if we can put them across in our journal after scientific studies with individuals and families, based on rational research methodology. Offer our old wine in new bottles.

### Need for research initiatives in developmental disorders

Let me now sum up my prefatory frustrations. The responsibility of psychiatrists who work with children to start original or serious replicative research studies is stupendous. This nascent sub-speciality of Child Psychiatry needs to examine ‘Developmental Disorders,’ including Learning Disorders, for research in a big way, because of the sheer force of the number of children in our country who suffer silently from these ‘silent handicaps’.

We need to look at these disorders from an Indian standpoint, and initiate independent research in their Psychopathology and Phenomenology, Nosology and Diagnosis, Epidemiology and Distribution, Etiology and Pathogenesis, and Methods of Management; furthermore, in major centers, their Genetics and Neuroscience. If major centers have this wisdom and the initiative, we need not become footsloggers in research.

## EDITORIALS AND PRESIDENTIAL COLUMNS

There is a recent spurt of interest in child psychiatry. T.S.S. Rao’s moving editorial in early 2008,[1] followed the release of Aamir Khan’s movie, ‘Taare Zameen Par’. Rao and Krishna have pondered over several facets of Learning Disorders, on the agony of the affected child, and the failure of the family and the schooling system to identify and manage these disorders. Today’s performance-oriented society too comes under their scrutiny, and they raise contemporary issues of adolescent suicide. I have, however, grave concerns when we point fingers solely at the pressure of the education system on children for academic achievement as the major cause for self-harm. The past three decades of experience have taught me to consider the present-day children’s inability to tolerate frustration and criticism as a major culprit in attempted suicides and not to merely condemn well-meaning parents and teachers, as the media is wont to do. In which case, non-hierarchical, permissive parenting, or autocratic parenting may be the malefactor. Disciplining children is not meant to be misinterpreted as punishment; discipline is to provide the skill of adaptability and resilience which eliminates any thought of deliberate self-harm. However, this needs appropriate research and evidence base.

P.C. Shastri never misses an opportunity to call upon our society and the medical fraternity to focus on mental health and basic education of children in our country. He has eloquently, using demographic data and extensive statistics, argued for a national comprehensive policy in his Presidential columns in the April and October 2008 issues of IJP.[3,4] His article on planning child guidance services in India envisages developing about 10,000 CGCs for the entire country to deal with developmental and other psychiatric disorders in children.

The Presidential address by P.C. Shastri on ‘Promotion and prevention in child mental health’ in January 2009,[5] elaborates on a National Action Plan for child mental health, based on the concept of a ‘single window operation’ in every district.

## IJP: CONCEPTUAL METAMORPHOSES ACROSS DECADES

After perusing the IJP articles of the 1980s on Developmental Disorders, which look at the mere psychosocial etiopathogenic concepts of developmental disorders, including ‘Learning Disability’, it is incredible to examine where the neurosciences of these disorders stand today.

In an investigation of 100 cases of learning disabilities by S. Khurana in 1980,[6] the implications were that causation of this difficulty was dependant on a disturbed relationship with the father, impaired relationship at school, fear of the teacher, fear of school, greater pressure of achievement on boys causing greater disability, disability being ‘essentially urban’, and that this disability and other problems such as enuresis and stammering ‘are results of traumatic experiences’ in the school, and so on and so forth. Bapna and Ramanujam, in 1976, had drawn similar conclusions emphasizing the parent–child relationship.[7]

Currently, every one of these assertions has been proved wrong by the exponential technology that peeps into live brain cells as they go about performing various mental functions.[8] Today, the concepts related to ‘hyperactive’, ‘hyperkinetic’, and ‘MBD syndromes’ appear as empiricisms in retrospect, when one reads through articles by P. Chawla[9] and Baldev K *et al*.[10]

Today, research using cutting-edge technology uncovers both the morphology and function of various parts of the brain, to reveal how psychiatric disorders occur in children. It enables us to comprehend how two dynamic processes – synaptic pruning and myelination – continue from early childhood through adolescence and keep modulating cognitive and social skills, and behavior.[11] It should be possible for research in developmental disorders in India to operate at this level, and for such research to be published in IJP.

### Intellectual Disability (Mental Retardation)

All developmental disorders tended to be ‘stigmatizing disabilities’. Our field has now moved to change the name of ‘Mental Retardation,’ replacing it with ‘Intellectual Disability,’ perhaps to de-stigmatize the term, and to give precision to the term.[12]

In India, considerable focus has been paid to Intellectual Disability (Mental Retardation) and its various aspects, although not much on its management.[13] Other developmental disorders such as Specific and Pervasive developmental disorders have not found much space in IJP, although the potential is enormous.

### Learning Disorders

Humans were not born to read, or to write.[14] Oral language skills originated a hundred thousand years ago, but reading as we know came about only a few thousand years back. If the creator did not allocate a place for it in the beginning, how did the brain then acquire it? Neuroplasticity, of course, is what made it possible, within whatever cells were given for the function of language. Multiple skills are involved in learning to read the ‘spoken sounds’ that get mapped into the left brain as ‘written symbols’ (letters) and thus as words that mean something. Reading comprehension, the process of understanding automatically as we read, is the extraction of meaning from written language.[14] This extraction of meaning through reading is not possible in children with learning disorders, especially reading disorder.

‘Listening’ comprehension, on the other hand, is excellent in the LD child, though he cannot ‘read’ and comprehend; he can answer the teacher’s question orally, but cannot write the same. Hence, the axiom that the ‘LD child would be the smartest lad in the whole school if instruction were entirely oral!’

From the neural findings, there is a current consensus among researchers that the central problem in Learning Disorders reflects a core deficit in the Language system.

Similarly, attention as in Attention-Deficit Hyperactivity Disorder (ADHD), was not seen as a deficit in Learning Disorders, until recently. Today several lines of evidence — behavioral, genetic, and neuroimaging studies — show that attention mechanisms play an active role in reading and reading comprehension.[14] Learning disorders, therefore, are not pure syndromes.

Our experience at Cochin, with over five thousand children assessed for poor school performance (PSP) on a hierarchical multidisciplinary model, has given us the strength to posit a ‘spectrum-construct’ of developmental disorders.[8] An unusually high degree of co-existence of all these developmental disorders together, along with conditions such as ADHD, obsessive / obsessive spectrum disorder, as well as a pattern of presentation that is increasingly showing a stereotype encouraged us to approach these disorders as a ‘mixed-bag’ initially, and now as possibly the ‘unitary-bag’.[15]

Our current understanding tells us that the neural processes involved in learning to read, write, spell, and perform maths themselves develop and change the brain cells both physiologically and functionally. Therefore, all the above academic skills are obviously neurally interconnected along with the core circuits in the language and attentional systems. We, therefore, need to look at the ‘connectivity deficit’ in the entire basket of developmental disorders, which percolates into our daily clinical practice. Concepts of these disorders have truly metamorphosed.

This explanatory note was to indicate that Learning Disorders cannot be seen in isolation, either for diagnosis or for management. Every learning disorder may not constitute a ‘disability’. Hence my title, ‘Learning and other Developmental Disorders’. Furthermore, we know here today that these disorders even respond to interventions, including pharmacological ones.

### Changes in operational definition of LD

A loosening of definition criteria for Learning Disorders (LD) seems to be taking place; the Federal definition of LD adopted by the US Office of Education (to determine eligibility for special education) has drastically changed the concepts.[14] These recent changes are of great relevance to psychiatrists.

The clinical definition of LD denotes a significant impairment in the acquisition and use of the academic skills of reading, writing, spelling, and arithmetic, in the background of the child having normal or above-normal intelligence. This connotes a discrepancy factor: a severe discrepancy between the child’s intellectual ability and his achievement on paper. At present, this definition also applies to it being life-long, assuming that there is no response to intervention (RTI). As against the DSM definition, two basic components have drastically changed now in the Federal definition:

- There is no requirement for the discrepancy factor anymore in the US Federal regulation, that is, there need not be a discrepancy between intellectual ability reflected by IQ and the child’s achievement, but,
- the federal regulation stipulates that the child be given quality intervention upon diagnosis; state sponsored Special Education will *only* be for those children who *do not* respond to this intervention (RTI)!

### Nosological shifts

In DSM III and DSM IIIR, all Academic Skills Disorders were classified under Axis II, they being developmental. DSM IV-TR now codes these on Axis I under clinical disorders. This may be quite significant for clinicians like us; the current definitions in DSM IV-TR recognize the multi-dimensional nature of these developmental disorders.

The other very significant shift in DSM is that of Communication Disorders (developmental disorders of language) and Motor Skill Disorders (developmental co-ordination disorders) from Axis II in DSM IIIR, to Axis I in DSM IV. I reckon this opens up greater pharmacotherapeutic and prognostic possibilities for clinicians who think like we now do.

### Child psychiatric epidemiology in India

A very significant attempt to synthesize available research on the prevalence of child psychiatric disorders, and highlight methodological trends was undertaken by P. Bola and M. Kapur[16] They evaluated 55 epidemiological studies conducted between 1964 and 2002, in the community and school settings. This one review made my job so much quicker and shorter, as most of the important epidemiological studies in this field had been covered by the authors. It even included my MD thesis work[17] that used, as in most studies, a Children’s Behavior Questionnaire (CBQ), and a clinical interview. As could be expected, the prevalence rates were very varied within the community-based and school-based studies. This meticulously written article is a lesson in critical appraisal of epidemiological studies.

I must use this occasion to pay tribute to the Late Prof. R.L. Kapur, and Dr. Malavika Kapur for the thorough, painstaking, and uncompromising methodology in their research studies; ‘The Great Universe of Kota’ is a living testimony.

The work of Malhotra S. *et al*., in epidemiological research methodology and in the development of instruments has been significant. Their recent study on ‘incidence of childhood psychiatric disorders in India’[18] is a longitudinal work, taking off from their well-known 2002 prevalence study. These studies have differentially diagnosed learning disorders.

## CONCLUSION

The core concepts about all developmental disorders seem to be drastically changing with the gathering evidence of genetic studies, with gene loci, pharmacogenomic studies, vigorous research in neural sciences, and evidence-based investigations for pharmacotherapy in these disorders. The multidimensional nature of these disorders is evident from research as well as clinical studies. The ‘spectrum-construct’ seems to enable pharmacological options for therapeutic intervention, along with the regular multi-pronged, non-pharmacological options.

This sojourn from the times of psychodynamic empiricism in child psychiatry to the current application of basic neurosciences to children’s day-to-day problems, from the twentieth century to the twenty-first century, has been remarkably worth its while. Now we require structured research and publication in our national Psychiatry journal. Long Live IJP!
